# Supplementary figures and images for: The Herpes Simplex Virus pUL16 and pUL21 Proteins Prevent Capsids from Docking at Nuclear Pore Complexes
Source: PLoS Pathog. 2023 Dec 1;19(12):e1011832. doi: 10.1371/journal.ppat.1011832 (PMC10718459; doi:10.1371/journal.ppat.1011832)

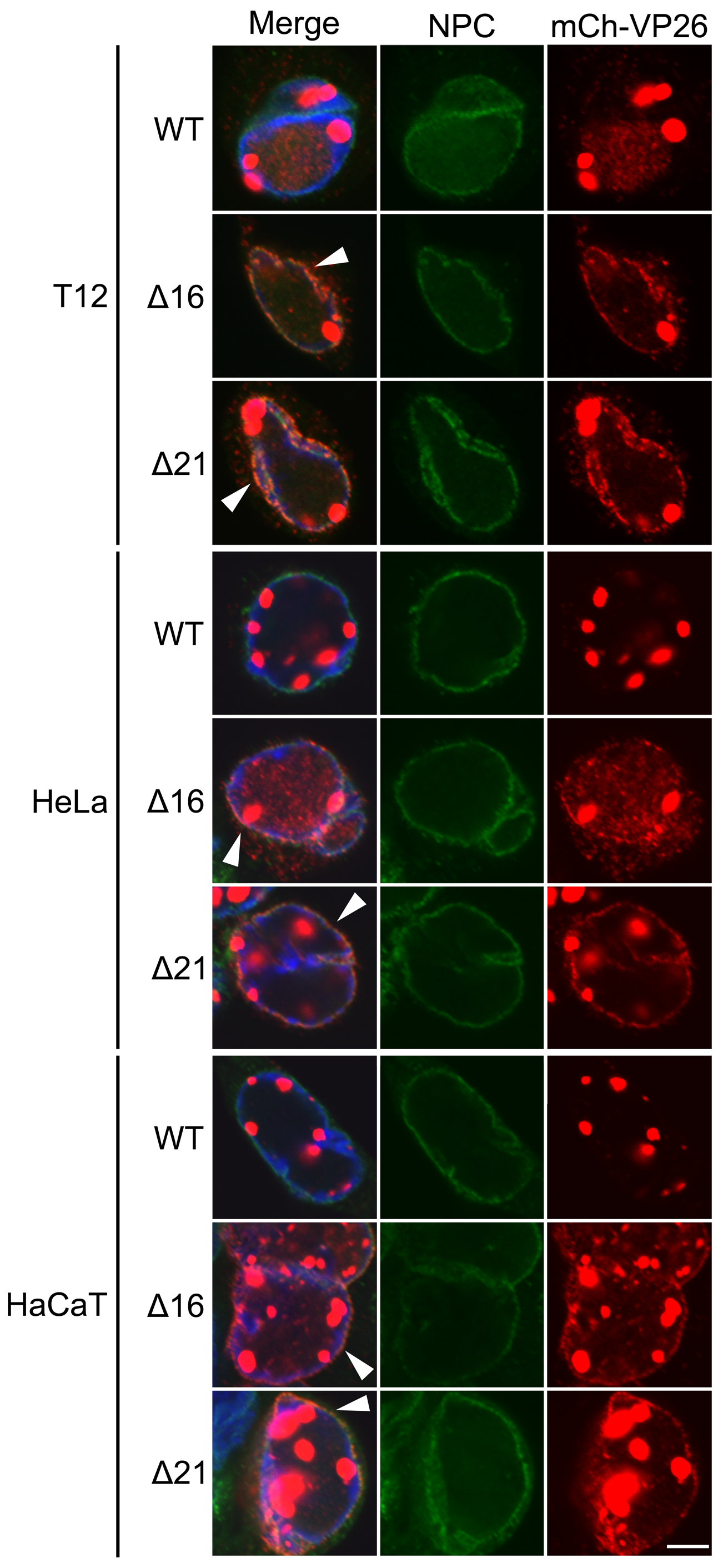

Supplement: S1 Fig — Cells were infected with HSV-2 186 mCh-VP26 WT, Δ16, or Δ21 virus at an MOI of 0.1 and fixed at 18 hpi. After fixation, cells were permeabilized with TX-100 and stained for NPCs. Representative confocal images of HSV-2 infected T12, HeLa, and HaCaT cells. Merge shows the overlay of Hoechst 33342 (DNA) (blue), NPCs (green), and HSV-2 capsids (red). The scale bar indicates 5μm. Arrowheads indicate cells with mCh-VP26 capsid fluorescence colocalized with NPC fluorescence. (TIF) [file ppat.1011832.s001.tif]

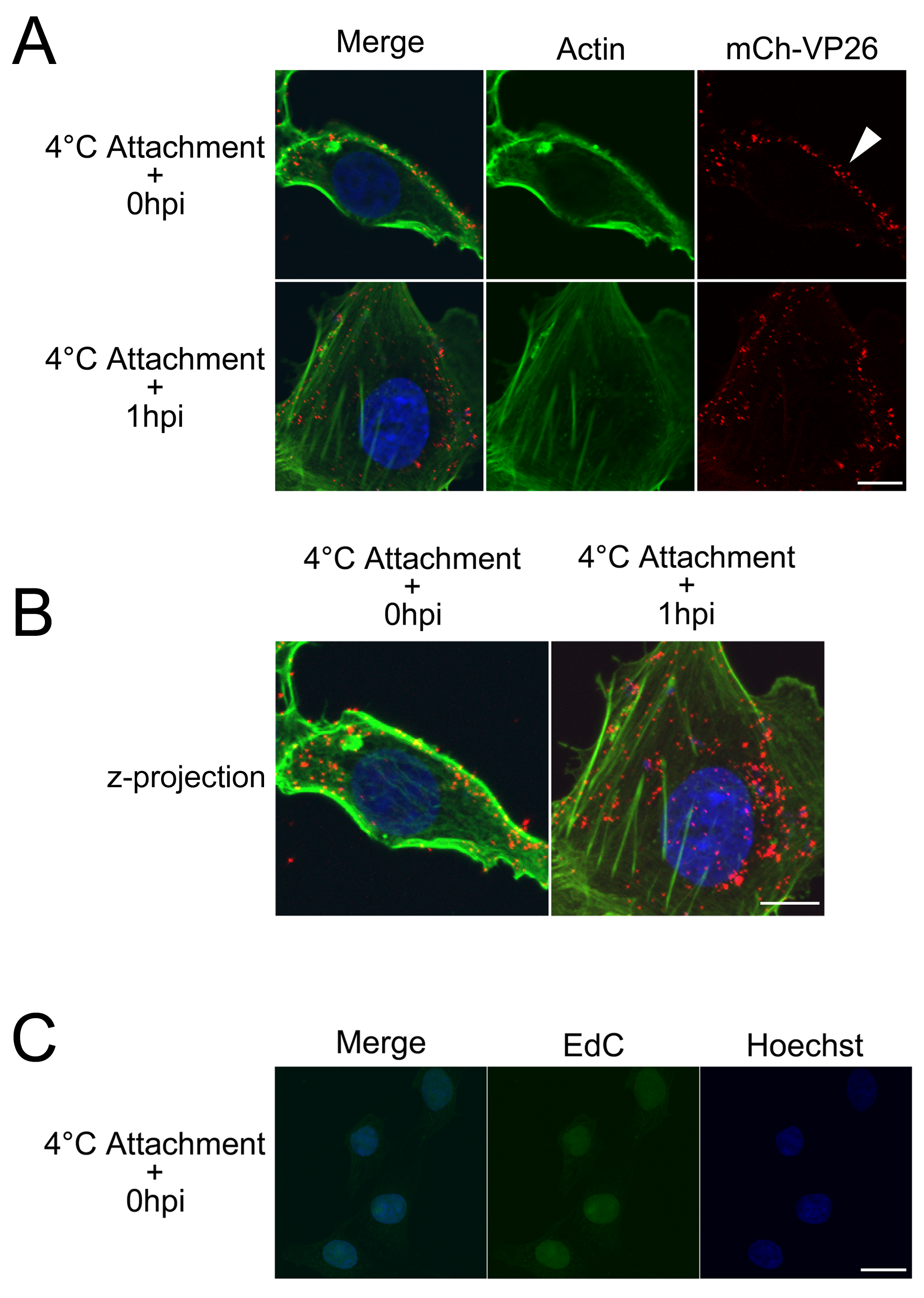

Supplement: S2 Fig — A) Representative confocal sections of Vero cells infected on ice with HSV-2 186 mCh-VP26 at an MOI of 3 for one hour and fixed immediately (0 hpi) or after being shifted to 37°C for one hour (1 hpi). Images show Hoechst 33342 (DNA) (blue), phalloidin staining of actin filaments (green) and HSV-2 186 mCh-VP26 capsids (red). The scale bar is 10μm. The arrowhead indicates mCh-VP26 capsids accumulated at the cell surface. B) z-projection images of the HSV-2 186 mChVP26 infected cells presented in panel A. C) Representative image of Vero cells infected on ice with HSV-2 186 WT EdC at an MOI of 3 for one hour and fixed at 0 hpi. Merge shows the overlay of Hoechst 33342 (DNA) (blue) and background Click-chemistry staining (green). The scale bar is 20μm. (TIF) [file ppat.1011832.s002.tif]

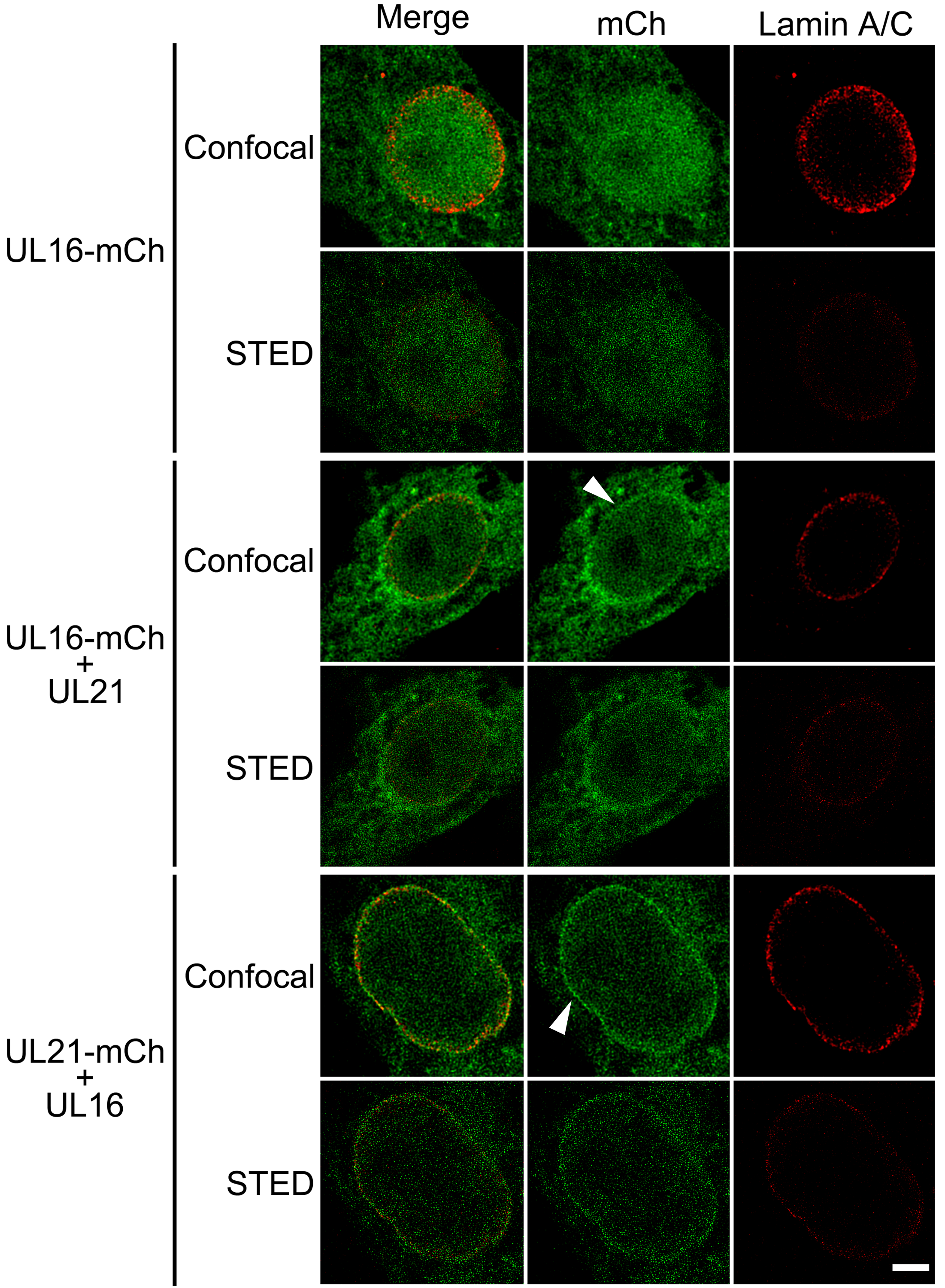

Supplement: S3 Fig — Cells were imaged by confocal microscopy and stimulated emission depletion (STED) microscopy. Cross section views of the nuclei are show in cells transfected with pUL16-mCh, pUL16-mCh and pUL21, or pUL21-mCh and pUL16 expression plasmids. Cells were stained with antisera against mCh and lamin A/C. The scale bar is 5μm. Arrowheads indicate cells with pUL16-mCh/pUL21-mCh nuclear rim staining. (TIF) [file ppat.1011832.s003.tif]
