# Supplementary material for: The Herpes Simplex Virus pUL16 and pUL21 Proteins Prevent Capsids from Docking at Nuclear Pore Complexes
Source: PLoS Pathog. 2023 Dec 1;19(12):e1011832. doi: 10.1371/journal.ppat.1011832 (PMC10718459; doi:10.1371/journal.ppat.1011832)
Supplement: Attachment — Submitted filename: Response to reviews_submitted.pdf [file ppat.1011832.s004.pdf]

Response to Reviews:

Responses to Reviewer queries are italicized.

Part II – Major Issues: Key Experiments Required for Acceptance

Reviewer #1:

1. all raw image data (image files with microscope metadata) used to quantify phenotypes should be deposited in repositories such as Dryad. Since I do not have access to the data that was used to generate figures 1B-D, 2D, 3B-C, 4B-C,E, 5B-C, E-F, and 6B, I cannot fully evaluate the validity of the claimed findings. See also https://plos.org/open-science/open-data/#accessible-data.

Response: Raw image files related to image quantification of Figures 2D, 3E, 4B-C, E, 5B-C, 6B, and 7E have been uploaded to Dryad. Data related to figures 1B-D and 3B-C were already shown in the figures 1A and 3A. The legends to these figures have been clarified accordingly. The data shown in 5E-F was quantified by scoring ICP27 and Us3 positive transfected cells by counting cells in numerous fields under the epifluorescence microscope. Details of how this was performed are shown in a modified legend to Fig 5.

2. some depicted image data is very hard to interpret in the supplied pdf, such as Fig. 4D. The authors should depict single channels in greyscale to make them easier to view. Again, also the underlying raw data for all figures should be made accessible for both the reviewer as well as the reader.

Response: Brightness and contrast has been uniformly adjusted in all images shown in Fig 4D. Nuclear EdC labeled puncta are now easier to see.

3. it is mostly unclear how image analysis was done for many figures. For example, the authors need to explain what is meant by Fig. 1 in line 875 "cell starting within the nucleus (distance 0) and extending into the cytoplasm". What point in the nucleus was defined as point 0? How was this made comparable between cells? How were the resulting curves from all cells averaged? Was any alignment done?

Response: This has been clarified in modified legends to Figs 1 and 3. Also see response to point 1, above.

4. Fig. 3A/B/C: The provided data indicates that capsids accumulate at the nuclear membrane where NPC is also. At this resolution, both signals often colocalize. However, this does not strictly indicate a direct association. More direct evidence is needed to claim that " pUL16 and pUL21 prevent incoming nucleocapsids from docking at NPCs," as stated in the abstract.

Response: The data shown in Fig 3ABC show accumulations of egressing capsids at the nuclear periphery, not incoming capsids, as stated by the Reviewer. The data that support the conclusion that " pUL16 and pUL21 prevent <u>incoming nucleocapsids</u> from docking at NPCs," as stated in the abstract can be found in Figs 4 and 5.

5. Fig. 3D: The phenotype in TEM (empty, docked capsids at NPCs) needs to be quantified, and the number of inspected cells needs to be stated if the authors wish to state "that HSV pUL16 and pUL21 deletion mutants accumulate empty capsids at the cytoplasmic face of NPCs late in infection."(lines 49-50).

Response: Agreed. These new data can be found in new Fig 3E. Numbers of infected cells examined are stated in the new legend to Fig 3.

6. Fig. 4D: It is unclear how the described experiment was performed. How do the authors exclude capsids that spilled their DNA in the cytoplasm?

Response: This experiment monitors viral genomic DNA, not capsids, as suggested by the Reviewer. DNA released into the cytoplasm would be detected by click chemistry as we have shown previously (PMID: 36374856). Only nuclear EdC signals were quantified as indicated in the y-axis of Fig 4E.

This signal might be above nuclei but is projected into the same slice. It is unclear which imaging settings were used to exclude this possibility (pinhole size, z-resolution). Also, were whole cells imaged in 3D, or were just single slices taken?

Response: The images shown are single confocal sections. The Reviewer's concern is not clear. Puncta with fluorescence intensities over background were scored. In response to Reviewer 3, point 10, below, we have included new controls in Fig S2C, that show background levels of click reactions in cells infected at 4˚C.

7. Fig. 6 C/D: the "colocalizing signal" indicated to prove the association of UL16 and UL21 with capsids are mostly aggregates and not single particles.

Response: Some signals appear to be capsid aggregates and some also appear to be single particles. The point we are trying to make is that the pUL21 and pUL16 EGFP fusion proteins have the capacity to interact with capsids. Further, the observation that pUL21-EGFP, but not pUL16-EGFP, interacts with nuclear capsid signals (consistent with the biochemical data of Wills and colleagues) suggests that the co-localizing EGFP signal is specific rather than non-specific "trapping" of EGFP fusion proteins within capsid aggregates.

8. Fig. 7: The claimed phenotypes need to be quantified.

Response: The data have been quantified in a new Fig 7E and the corresponding images used for quantification uploaded to Dryad.

9. Fig. 8/9: A control labeling of the inner nuclear membrane is needed if the authors wish to claim that UL21 and UL16 localize to the outer nuclear membrane. The resolution of STED microscopy might not be sufficient alone to determine if a protein is on the inner or outer nuclear membrane. The distance between the two nuclear membranes is 30-50nm. The resolution of a STED microscope typically ranges around 50nm. Antibodies have a size of 10nm and fluorescent proteins 5nm. Summing up these sizes means that the localization precision may not be sufficient for the author's claims. Immuno-EM might be an alternative approach.

Response: The Reviewer is correct. We are unable to unambiguously distinguish whether pUL16 and pUL21 localize specifically to inner or outer nuclear membranes. Additional new data, provided in new Fig 8A, shows some co-localization of pUL21 with the nuclear lamina marker, lamin A/C. Indeed, it is likely that these proteins localize to both inner and outer nuclear membranes. Our descriptions of pUL16 and pUL21 localization have been revised accordingly throughout the text. Importantly, these observations have no impact on the central conclusions made in this manuscript.

Reviewer #2:

1. In Fig 2A, the differences between WT and Δ16 or Δ21 is not obvious, please state the reason.

Response: A "ring" of capsid signal is evident around nuclei of Δ16 and Δ21 infected cells that is not seen in WT infected cells as indicated by the arrowheads. See also figures 1A, 2B, C, 3A, 6C, D and 7C, D for similar findings.

2. The authors should perform some experiments in the epithelial cells, such as HFF or HEL.

Response: We have examined Vero (green monkey), HeLa and human keratinocytes (HaCaT), that are particularly relevant to HSV infection. Therefore, our observations do not appear to be cell type or species (human vs green monkey) specific. Nor are they specific to HSV species. Nevertheless, we have performed new experiments in life-extended human foreskin fibroblasts and included these data in a new Fig S1.

Reviewer #3:

1. The introduction to this work is a quite simplistic overview of HSV replication and exit pathways and reads a little bit like a student project summary. Considering this is about the function of UL16 and UL21, the introduction could do with a much better detailed and relevant introduction to these proteins and any previous data on their roles and importance for overall replication. This links to point 2.

Response: Our Introduction was meant to provide the necessary background information required to contextualize the key findings made in this manuscript as succinctly as possible and to a broad audience. We think we succeeded. The Introduction was not meant to serve as a review of the broad range of activities reported for pUL21 and pUL16 that, for the most part, have little relevance to this study. Nevertheless, we have introduced the pUL16 and pUL21 mutant strains, provided citations to papers where they have been characterized, and indicated that they all suffer replication defects in a modified Introduction.

2. The first phenotype that we are presented with for the deletion mutants for UL16 and UL21 is on the localization of the major capsid protein VP5. There is no data on the effect of these deletions on overall replication of the virus and if this has not previously been published upon by the authors then clearly this would be amongst the first most relevant data to present. Conversely if they have reported previously on the effect of these deletions on overall replication then that obviously should be introduced. But perhaps more relevant would be the preliminary discussion of the effect of deletion of UL16 and or UL21 not only on overall replication of HSV, but on the effect on extracellular versus cytoplasmic infectious virus. There could be an effect on both, there could be an effect only on cytoplasmic infectious virus. There could even be a result that, despite the validity of preventing re-engagement of progeny virus with the nuclear pore, this actually made a minor impact on the overall abundance of released virus. Alternatively, if the deletion mutants each essentially abolished replication having a profound impact on the production of infectious virus, either way the relevant data should be either introduced and or presented early.

Response: This is not the case. Figure 1 shows the localization of mCh-VP26 fusion proteins produced by several recombinant virus strains. These deletion strains have been published and characterized previously and the citations included in the original manuscript in the Cells and Viruses section of the Materials and Methods. We have altered the Introduction to indicate that all of the pUL16 and pUL21 mutants have replication deficiencies.

3. The first data we're given is on the distribution of a fluorescent fusion protein mCh-VP26, (a structural capsid protein), in cells infected with wild type virus versus the deletion mutants. The key phenotype here is on the distribution of mChVP26 to the outside nuclear rim. In the text, (page 5, line 132/133), this is described as ..” An intense ring of mChVP-26 Fluorescence at the nuclear periphery.” While there does appear to be a phenotype with increased nuclear rim localization in this and other figures, this localization can appear rather patchy and certainly not in an intact intense ring. For example, even in the first figure for the UL21 deletion mutant the fluorescent fusion protein is mainly located in very large aggregates inside the nucleus, which themselves seem rather variable throughout the paper, with a minor population at the nuclear rim, and in patches. The same is true for the mutants in figure 2A and the HSV 1 versions in figure 2B and elsewhere. This may well be the proposed phenotype but perhaps it should be more accurately described, and this leads to another point on the way it is classified and quantified.

Response: We have modified our description of this phenotype from “intense ring” to “aberrant accumulation”.

4. The authors state, page 7, line 166, “The percentage of sales with an abundance of capsids at the cytoplasmic face of the nuclear envelope was quantified”. This is then given as a percentage of nuclei, i.e., a single nucleus will be scored positive or not positive. But what exactly this means is not clear. For example, in many of the images it is not the case that majority of the capsid signal is at the nuclear envelope, in many cases it is clearly within the nucleus

and in several in large nuclear aggregates. This may ultimately be an understandably difficult classification, but some more accurate description of the classification could be given.

Response: The purpose of these experiments was to score the capsid signal on the cytoplasmic face of the nuclear envelope, not the capsid signal within the nucleus. Line 166 refers to data in Figs 2C and 2D where there is no nuclear capsid signal as these cells were permeabilized using saponin.

5. In this vein the authors have an opportunity to strengthen the interpretation of the phenotype from their transmission electron microscopy in figure 3. They suggest, page 8, line 187, that for the wild type virus at 18 hours after infection it was "very rare" to see capsids docked at the nuclear pore, while this was "common" for the UL16 and UL21 mutant viruses. This could also be quantified, and indeed normalised; for example number of NPC docked capsids/total cytoplasmic capsids, for each of the wild type and mutant viruses. This would then support and supplement the classification from the immunofluorescence studies.

Response: Agreed. These new data can be found in new figure 3E.

6. In Figure 4 the authors then use the idea of superinfection exclusion to support their proposition. The idea is that prior infection with wild type HSV would, by virtue of its expression of UL16 and UL21, block the nuclear docking and genome entry of a subsequent super-infecting virus. On a minor point the description of this experiment in the legend to Figure 4 seems at odds with the description given in materials and methods. In the legend the Superinfecting virus is stated as being at an MOI of 1, while stated as an MOI of three in the materials and methods. In the legend the analysis of the Superinfecting virus is at one hour after infection, while in the materials and methods, superinfection is for one hour on ice followed by a shift to 37 degrees and subsequent analysis two hours later. While this might seem minor the detail is important for the following points.

Response: An MOI of 1 was used for the initial infection and an MOI of 3 was used for the superinfection. The analysis of the superinfection was performed at 1 hpi. This has been corrected in the figure legend to Fig 4 and in the Materials and Methods.

7. Figure 4a includes a normal infection of wild-type into previously uninfected cells (mock). The numbers of capsids on the cell appeared to be in the hundreds, and even with a relatively poor particle infectivity, this seems a very high particle count.

Response: There is no query here. See response to comment 8, below.

8. In this regard these are maximum projections and thus very likely will measure viruses/capsids bound to the outside of cells (indeed that is why there is such a very high particle count). Given that, following the Superinfection and shift to 37 degrees for two hours, the authors propose their examining internalised capsids. How do they know? There are no controls. Minimally the authors should superinfect for the one hour on ice, and fix and sample there, without any shift up in temperature. It may well be that a quite a high proportion of what they measure is at the cell surface reflecting virus that has not or will not enter cells.

Response: We have performed these controls that are provided in new supplemental Fig S2A, B. When fixed immediately after 1h at 4˚C the majority of capsids are seen at the cell surface, which is strikingly different from what is observed when cells have been shifted to 37˚C for 1h.

9. Having said that the authors are not claiming any significant phenotype of primary infection by the mutant viruses in affecting the cytoplasmic entry or localization to the nuclear periphery of the Superinfecting virus.

Response: This is correct.

10. What they do claim is that there is a phenotype in allowing nuclear pore docking and genome entry into the nucleus of the Superinfecting virus. The idea is that a primary infection by a wild-type virus suppresses genome entry of the Superinfecting virus, while a primary infection by either of the UL16 or UL21 mutant viruses has much less of an effect in this regard, thus permitting significantly more genome entry of the Superinfecting virus. However the imaging data are not particularly clear and no controls are presented. The images are proposed to represent uncoated EdC-labelled genomes entering the nucleus after super infection. Quantitation of this indicates that under conditions where the primary infection is by wild-type virus, for the super-infecting virus there would be approximately one genome observed for every two cells on average. When the primary infection is by the UL16 or UL21 deletion mutants, then for the super-infecting virus there is approximately 1 genome for every one cell (or very slightly more). Notwithstanding the numbers of cells stated for the quantification, this seems a very marginal effect that is compounded by difficulty interpreting the images themselves. While there are some new clear foci that are proposed to represent uncoated nuclear genomes, there is also quite a lot of both diffuse membrane-like cytoplasmic and diffuse nucleoplasmic signal including what looks to be nucleolar signals in some cells. It would be useful to have some controls, even if presented a supplementary information, for example examining cells by the click chemistry reaction but where infection was only at 4 degrees, thus preventing entry and nuclear uncoating. Perhaps the background would remain but the nuclear foci it would disappear confirming their interpretation. Another piece of supplementary information that might influence the design of the assay would be showing increasing numbers of nuclear foci with multiplicity of infection of the super-infecting virus. However it might be easier to demonstrate the point with a different assay. The quantitation indicates that, Where the primary infection is wild type, only one in two cells would be superinfected. While where the primary infection is a mutant virus, all cells will be super infected. perhaps an easier and justice robust assay would be measuring very early protein expression, for example ICP4 or ICP0, if necessary using a fluorescent- fusion protein to discriminate the superinfecting virus. Another example of an experiment that would help support the proposal, is it the abundance of expression of UL16 and UL21 from the primary infection would correlate The decrease in the efficiency of super-infection. it might be possible to examine this, either by the nuclear genome entry assay or by the superinfecting protein expression assay.

Response: We agree that "examining cells by the click chemistry reaction but where infection was only at 4 degrees, thus preventing entry and nuclear uncoating. Perhaps the background would remain but the nuclear foci it would disappear confirming their interpretation. " is a great suggestion. We have done this and included these new data in new supplemental Fig S2C. Importantly, no EdC labelled fluorescent puncta were observed under these conditions.

11. In figure 5, pursuing the idea that prior expression of UL16 or UL21 would interfere with super infection, the authors ask topically express these proteins and then measure the influence on capsid recruitment to the proximity of the nuclear envelope. While in figure 4A the similar experiment shows extremely high numbers of capsids at various distances and locations in the infected cell, it is not clear why in the similar experiment in figure 5, there appeared to be very low numbers come on a few at most during infection of in this case the previously transfected cells. Nonetheless the experiments should have a very similar framework.

Response: It is not clear what the Reviewer is asking. What is "authors ask topically express"? What does "very low numbers come on a few at most during infection of in this case the previously transfected cells." mean? We think that the Reviewer is wondering why fewer capsids appear to be present in Fig. 5A versus Fig. 4A. The likely explanation for this is that 4A is a z-projection image of multiple sections whereas 5A shows single optical sections.

12. The paragraph, page 13 line 292, is a little confusing. it starts by confirming that UL16 and UL21 can interact with capsids, and finishes with the conclusion that UL16 and UL21 do not interact with incoming capsids, and thus are unlikely to prevent capsid recruitment to the nuclear pore. This needs a little better clarification and perhaps at this point more explicit proposal of what they are likely to do in preventing superinfection.

Response: The data, and the paragraph in question, clearly indicate that at <u>late times</u> post infection (i.e. egressing capsids/capsids assembled in the nuclei of infected cells) ectopically expressed pUL16-EGFP and pUL21-EGFP can interact with capsids. However, if cells are <u>ectopically</u> expressing pUL16-EGFP and pUL21-EGFP, <u>incoming</u> capsids (that are initiating infection) do not associate with the ectopically expressed pUL16-EGFP and pUL21-EGFP.

13. The experiment shown in figure 7 or actually some of the better images presented showing the difference in capsid protein recruitment to the nuclear rim by the mutants versus very little observed for the wild type virus.

Response: There is no query here.

14. In Figure 8 the authors use STED for more refined imaging analysis of the distribution of UL21 with regards to the nuclear envelope and in particular whether there is a significant relative accumulation on the cytoplasmic face. There are particularly convincing data here especially for the Cross sections with the MAB414. it would be useful to know how it was defined that they were examining the cytoplasmic face, and might not a Z- projection be useful in this regard. an obvious question also is whether they or indeed anyone else has reported on the nuclear rim localization of UL21 using antibody to endogenous protein.

Response: See response to Reviewer 1, query 9. Yes, we (Le Sage et al PMID: 23487471) and others (Graham and colleagues, PMID:34398933) have reported nuclear rim localization of pUL21 in HSV-2 and HSV-1 infected cells, respectively, using antisera reactive against pUL21.

15. With regards to Figure 9 the proposal for some population of UL16-cCh residing specifically at the outer nuclear rim, would be enhanced by the appropriate control of UL16-mCh alone, especially by STED, from where they are proposing a lattice like appearance. It is odd that the internal UL16 and indeed UL21 do not appear to be visualised in the cross section. is there an explanation for this and in particular when they do visualise the nuclear internal proteins do they also appear as a lattice like pattern in the STED. If there is a clear qualitative difference this would reinforce their case.

Response: The internal nuclear UL21 signal is very weak in the cross sections and not resolved in the STED. In the presence of UL21, the internal nuclear UL16 signals is also very weak and not resolved in the STED. As suggested by the Reviewer, we performed new STED experiments of pUL16 alone. These data are found in a new Fig. S3. Here, the internal nuclear pUL16 signal is greater (because no pUL21 is around to recruit it to the nuclear envelope). This signal was resolved in the STED, but did not show a lattice like appearance.

Part III – Minor Issues: Editorial and Data Presentation Modifications
Please use this section for editorial suggestions as well as relatively minor modifications of existing data that would enhance clarity.

Reviewer #1: -Fig. 4B would be easier to read if expressed in percent of mock.

Response: The Reviewer's rationale for this modification is not provided. We respectfully disagree.

-The number of analyzed cells for Fig. 3B and C is missing.

Response: Done. n=10 individual cells examined per condition.

- line 515-516: The ΔpUL16/pUL21 virus was grown on a 1:1 mixture of pUL21 and pUL16-complementing cells. These conditions could lead to a mixed population of viruses in the stock produced.

Response: Maybe. These conditions enabled us to prepare stocks of this strain. We have not performed single particle analysis. There is no query here.

- In lines 570-572: Duration of treatment of Nocodazole missing in the methods.

Response: Done. Information has been added to the figure legend and the Materials and Methods.

- In lines 620—622, it is hard to follow which plasmids were transfected together.

Response: We have modified this passage to improve clarity.

Reviewer #2: No

Reviewer #3: point 1 above

Response: Done. See response to point 1, above.
