# Supplementary material for: The Herpes Simplex Virus pUL16 and pUL21 Proteins Prevent Capsids from Docking at Nuclear Pore Complexes
Source: PLoS Pathog. 2023 Dec 1;19(12):e1011832. doi: 10.1371/journal.ppat.1011832 (PMC10718459; doi:10.1371/journal.ppat.1011832)
Supplement: Attachment — Submitted filename: Response to second review_final.docx [file ppat.1011832.s005.docx]

**Response to reviews:**

Reviewers 1 and 2 had no concerns. Below, please find our response to Reviewer 3. Our responses are in italics.

**Reviewer #3:**

The authors have gone some way to addressing the key points raised in response to the original manuscript. However certain points have not been adequately addressed or require further clarification.

**1.** With regards to original points 1 and 2, on providing some more and relevant introductory detail: While it might be case that a generalised background helps context, what would certainly help context, relevant to the details of this paper, would be more introductory information specifically on UL16 and UL21. Also stated, if the authors are interested in context, it would be extremely useful to mention in more detail on the extent of the impact of UL16 and UL21 deletion mutants, on replication, especially on intracellular versus extracellular yield. Their response to this point it really pays it lip service and doesn't serve the reader well when they state o the mutants, line 115, “to varying degrees, all of these strains suffer defects in virus replication”. Although they also point to the materials and methods indicating that some detail had been given there, in fact this simply refers back to the previous papers again. Referring back to previous papers in this way without more information as requested really doesn't aid context for the reader. They should give a succinct and clear statement of the extent of the phenotype on replication of intracellular and extracellular virus of these mutants, including differences in eg HSV-1 and HSV-2, to provide the context they apparently wish to provide.

*Response: The reviewer has requested that we emphasize information on the yield of “intracellular” versus “extracellular” virus for pUL16 and pUL21 mutant strains. Mbong and colleagues, studying HSV-1 pUL21, showed delays in the appearance of both intracellular infectious virus and extracellular virus that were attributed to a delay in the expression of virus gene products. Reductions in overall pUL21 mutant virus yields were also observed in this study with a roughly 5-fold reduction in end point extracellular virus and a 10-fold reduction in extracellular virus produced. By contrast, Muto and colleagues saw no replication deficiencies for their HSV-1 pUL21 mutant. Compounding the difficulty in interpreting the observations of Mbong, Muto and others (i.e. Baines et al 1994) relating to replication analysis of pUL21 mutants is the observation that mutant pUL21 strains that are not passaged on complementing cells rapidly acquire second site suppressor mutations that enhance their replication (Sarfo et al 2017, Benedyk et al 2021). The mutants reported in the Mbong, Muto and Baines studies were not passaged on complementing cells. Starkey and colleagues, studying HSV-1 pUL16 mutants, noted 10-fold replication deficiencies in both intracellular and extracellular virus replication.*

*Comparison of intracellular versus extracellular virus produced is particularly useful for identifying virus mutants that have “release” defects i.e. virus strains that accumulate intracellular infectious virus and release much less virus into the medium. In relation to the present study, it is not clear, how such an analysis is relevant insofar as one might expect that the consequences of a significant fraction of cytoplasmic nucleocapsids delivering their genomes back into the infected cell nucleus would be an overall reduction in both intracellular and extracellular virus, rather than a specific reduction in intracellular virus production, for example.*

*As requested, we have provided a comprehensive description of HSV pUL21 and pUL16 mutant phenotypes and putative protein functions in the Introduction. However, we have not gone into details about intracellular versus extracellular virus production for the reasons stated above. See lines 126-164 of the resubmitted manuscript for the newly added text.*

**2.** The original point, (4), referred to figure 2C and its quantitation in figure 2D. This latter figure assesses VP5 localisation as a proxy for capsids, and is plotted as ” nuclei with capsids surrounding the nuclear envelope”. This was given as a percentage of total nuclei. The query was on clarification of exactly what was being measured and how. The authors have now stated that this measurement solely concerns the analysis performed with saponin permeabilization for imaging, i.e. where there is no background VP5 detected in the nucleus. However, some clarification is still required. For example, in figure 2C, zoomed panels are illustrated presumably to help explain what the authors are scoring as positive and negative. With regard to the measurement, “nuclei with capsids surrounding the nuclear envelope”, they score the single image in figure 2C as negative for the wild type, but the image for the deletion mutants as positive. At least to this reviewer I cannot see the difference. The arrowheads added to the panels for the mutants aren't really explanatory and it is unclear why this is classed as different from the wild type image. More clarification is still required.

*Response: We have modified figure 2C to include a higher magnification inset that illustrates the differences between WT and mutant strains more clearly. Removal of the Hoechst channel from these images has also aided in distinguishing cells with and without VP5 signal at the nuclear periphery.*

**3.** A related point relates to the quantitation of these images, -and the proposal that the lack of UL16 or UL21 allows this capsid recruitment to the nuclear pore/periphery. From the quantitation this phenotype appears to be minor. While it they state that the assay has essentially no background, in other words, no wild type infected cells appear to show cytoplasmic VP5 in proximity to the nuclear envelope, nevertheless from the data, only approximately 1% of the single mutant viruses show this phenotype and are scored positive. Thus 99% of the mutant infected cells do not show capsid accumulation at the nuclear periphery. If this is the correct interpretation of their data, the authors should comment on this. Alternatively, if this is not the interpretation the authors wish to leave, they should clarify.

*Response: The Reviewer has misinterpreted the values shown in the graph. Less than 1% of WT show this capsid distribution. The graph clearly shows that ~7% of pUL16 and pUL21 single mutants show this and ~14% of the double mutant infected cells show accumulations of VP5 signal on the cytoplasmic face of the nuclear envelope.*

**4.** With regards to figure 5D, the authors indicate that prior ectopic expression of UL16 and UL21, results in a decreased expression of ICP27 from an incoming virus, despite there being detectable incoming capsids in the cytoplasm. This is apparently shown on the inset for figure 5D. However, it is not readily apparent on the figure what they are referring to, nor is there anything readily visible, or pointed to with arrows, in the corresponding mCH-VP26 channel. The authors should clarify and label very precisely with appropriate arrowheads.

*Response: As stated in the figure legend to Fig. 5 (lines 1167-1168) the arrows point to cells that express ICP27 whereas the arrowheads point to cells that do not. The inset shows a cell indicated by an arrowhead (i.e. that does not express ICP27) that clearly has red capsids within the cytoplasm. We have further clarified the legend to figure 5D by including the following “The inset image shows mCh-VP26 capsids inside a cell, that does not express ICP27 and has been co-transfected with pUL16 and pUL21 expression plasmids.” on lines 1168-1170.*

**5.** In figure 6 the authors indicate that while independent expression of UL16 shows a broad heterogeneous pattern of localization, co-expression with UL21, results in, “pUL16-GFP also concentrated at the nuclear envelope”. This, as with several other statements in the results, is an overemphasis: rather that a minor population of overall UL16 appeared to localise to the nuclear rim when co-expressed with UL21. The authors should correct the description that UL16 concentrated at the nuclear envelope.

*Response: Done. See lines 338 and 412 for revised descriptions of pUL16 localization to the nuclear envelope.*

**6.** In the same experiment the authors indicate that transfected and ectopically expressed UL16-GFP is recruited to and co-localises with cytoplasmic capsids produced later after w/t infection of the transfected cells Fig 6c, w/t). They indicate this with an arrow on the corresponding GFP panel. it is however extremely difficult to determine what is the capsid signal they are indicating that colocalises with this arrowed UL16-GFP region. It is not indicated on the mCH-VP26 panel, and I can determine very little mCH-VP26 signal there. In fact there doesn't seem to be much of a cytoplasmic capsid signal at all, rather the mCH-VP26 fusion protein appears to be in large nuclear aggregates. And there is certainly a lot of the UL16-GFP green signal without any VP26 association. The authors should clarify the figure and indicate the co-localising capsids much more precisely and clearly.

*Response: Done. Arrows are now present in EGFP and mCh-VP26 channels. The original resubmission showed arrows only in the merged panels*

**7.** Line 306, clarification needed. After the results indicating that ectopically expressed pUL16 and pUL21 can be recruited onto late assembling capsids the authors state in the same paragraph, “Collectively, these data suggest that , when ectopically expressed, pUL16 and pUL21 are not loaded onto incoming capsids “. But this mainly refers back to the previous paragraph and Figure 6a/a. For clarity the authors should state “Collectively, the data of Figure 6, suggest that , when ectopically expressed, pUL16 and pUL21 are not loaded onto incoming capsids…….”

*Response: Done. See lines 349-352 of modified manuscript.*

**8.** (Original point 14). With reference to pUL21 localisation in Figure 8, the query was whether other pertinent previous data on UL21 were available. The authors respond that there are, but they don’t include them at the appropriate juncture, nor indeed cite the relevant information from e.g. Benedyk et al. For example it would improve this paper if the authors acknowledged both their own and others work relevant to figure 8 by initially stating something like, line 339, “previous work from our own and other laboratories has demonstrated that a population of UL21 exhibits a nuclear rim like localization (refs). We next wished to pursue a more refined analysis of both UL21 and UL16 localization and in particular more precise localization with regards to the cytoplasmic and or nuclear face of the nuclear envelope” or something similar.

*Response: Done. See lines 383-385 of modified manuscript.*

**9.** Finally, coming back to the first point on putting this study in context, the authors state that their very basic description of the HSV life cycle, is to put the study in context for the reader. I suggest that readers of the manuscript will likely have a reasonable knowledge to the herpesvirus life cycle and that the reader would be better served by giving context regarding previous data not only on the extent or otherwise of any deficiency in actual virus replication (eg no defect for HSV-1, versus essential for HSV-2), any inconsistency in those phenotypes, but also information from previous publications from others (and indeed their own) on possible roles of these proteins. For example, pUL21 is reported as being required for efficient capsid nuclear egress, secondary envelopment and virus spread. But the requirement for pUL16 and pUL21 in nuclear egress varies depending on strain and cell type. In addition to promoting capsid translocation, the pUL21:pUL16 complex may promote wrapping of cytoplasmic virions via interactions with other viral proteins. pUL21 may also promote virus spread, via an interaction with gE. pUL21 has also been observed to promote the formation of long cellular processes, potentially by regulating microtubule polymerisation. It has also been reported to be an adaptor of protein phosphatase an affects phosphorylation of both cellular and viral nuclear egress proteins. Perhaps not all of this is related to this present study, but certainly some attempt should be made, instead of a rather basic introduction, to give context with information both on the effect of deletion of these proteins on virus yield (but more than a cursory back reference without stating the actual result) and prior art of multiplicity of functions of these proteins.

*Response: Done. See response to Reviewer point 1, above. See lines 126-164 of the resubmitted manuscript for the newly added text.*
